# Interventions to prevent youth violence in Latin America: a systematic review

**DOI:** 10.1007/s00038-016-0909-6

**Published:** 2016-10-20

**Authors:** Erika E. Atienzo, Susan K. Baxter, Eva Kaltenthaler

**Affiliations:** School of Health and Related Research (ScHARR), University of Sheffield, Sheffield, UK

**Keywords:** Violence, Youth violence, Preventive interventions, Latin America, Systematic review

## Abstract

**Objectives:**

This review aims to summarise evidence on the effectiveness of interventions to prevent youth violence in Latin America.

**Methods:**

A systematic search on 13 academic databases was conducted to locate studies evaluating a primary or secondary prevention intervention in Latin America. Studies could use any type of quantitative design to assess outcomes related to youth violence. A search of websites, references and citation searching was also carried out. The quality of each study was assessed.

**Results:**

Nine studies were identified. Most documented positive effects of the interventions on the perception of youth violence present in the community/school. Evidence was found of a reduction in homicides and juvenile crimes in three studies, two of which evaluated a community-based intervention. There were mixed results for the self-report of participation on violent acts. The majority of the studies lacked of a rigorous design.

**Conclusions:**

Most of the interventions had some promising results, including the reduction of homicides within communities. Community-based programmes were the most consistent regarding an effectiveness to prevent violence. However, the evidence for Latin America is still scarce and relies on non-rigorously designed studies.

**Electronic supplementary material:**

The online version of this article (doi:10.1007/s00038-016-0909-6) contains supplementary material, which is available to authorized users.

## Introduction

Youth violence is a global problem. Every year, around 2.5 % of the registered deaths are due to violence, and among these, almost half occurs in young people (WHO 2014). It has been estimated that around 200,000 youth aged 10–29 years are murdered each year (WHO 2015). Violence among young people imposes a high cost to health services, reduces productivity and affect the functioning of essential services within the community (Mercy et al. 2002). High levels of violence might also stigmatise neighbourhoods, hinder investment and reduce social cohesion (Willman and Makisaka 2010).

According to the World Health Organization (WHO), youth violence can be defined as a form of community interpersonal violence, which is that inflicted by an individual or small group on other people who are not relatives (Dahlberg and Krug 2002; Hall et al. 2012; WHO 2014). Although the definition of young people includes individuals aged 10–29 years (Mercy et al. 2002), many of the preventive efforts for juvenile violence targets people aged 10–24 years (Hall et al. 2012).

The global rate of intentional homicides in 2013 was estimated to be 6.2 per 100,000 population, with 16.7 victims per 100,000 men aged 15–29 years and 3.8 among young women (UNODC 2014). However, non-fatal interpersonal violence occurs more frequently than homicide, and may also have lifelong consequences (WHO 2014, 2015). Thus, other less serious forms of violence such as attacks, threats, injuries to other persons, physical fighting, bully, discipline problems and other violent or non-violent crimes are alternative indicators of youth violence (Basch 2011; Matjasko et al. 2012).

While violence is recognised as a problem internationally, regional differences in the levels of violence have consistently been reported. Rates of murders among young men aged 15–29 in South and Central America are up to four times higher than the global rate for this age group (UNODC 2014). Traditionally, Latin America has been recognised as one of the most violent regions (Moser and van Bronkhorst 1999; Peetz 2011), with most of the homicides in the population occurring as a result of interpersonal violence, drug-related crimes and juvenile gangs (Cohen and Rubio 2007; Heinemann and Verner 2006; Imbusch et al. 2011; Moser and McIlwaine 2006; Peetz 2011; United Nations 2007). In addition, the phenomenon of school-based violence and bullying is on the rise (Cunningham et al. 2008; Felix et al. 2011). Yet, there is little knowledge on the effectiveness of programmes to prevent violence both in the general population and in youths in Latin America (Ardila-Gomez et al. 2015; Heinemann and Verner 2006; Moestue et al. 2013).

There are many published reviews on the effects of different types of programmes on the prevention of juvenile violence throughout the world; e.g. school-based interventions (Hahn et al. 2007; Mytton et al. 2002, 2006; Oliver et al. 2011; Wilson and Lipsey 2007); after-school programmes (Durlak and Weisberg 2007; Kremer et al. 2015); community programmes (Tolan et al. 2008; Wilson and Lipsey 2000); training to parents (Bilukha et al. 2005; Maughan et al. 2005; Piquero et al. 2008); and other types (Hahn et al. 2005; Limbos et al. 2007; Petrosino et al. 2013; Weinstein et al. 2014).

According to an international meta-review (Matjasko et al. 2012), 52 systematic reviews and meta-analyses on the effectiveness of primary, secondary or tertiary prevention strategies for the prevention of youth violence were published between 1950 and 2009. However, the vast majority of programmes have been implemented in the U.S., Canada, the U.K., Australia or other English-speaking countries, while interventions evaluated in developing regions are still rare (Limbos et al. 2007; Office of the Surgeon General (US) et al. 2001; Willman and Makisaka 2010; WHO 2010, 2015). The sub-representation of research from developing countries means that recommendations from previous syntheses are based on what has been effective in high-income countries. This is problematic since, to be effective, preventive strategies need to be context-sensitive. Matching programmes to the targeted population is a core element in successful prevention programming (Nation et al. 2003).

In Latin America, factors influencing the origins of youth violence are related to social conditions present throughout the region such as high levels of inequality and poverty, a lack of quality education, a culture of masculinity that promotes the involvement in conflict, urban growth and a drug-trafficking context (Heinemann and Verner 2006; Moser and van Bronkhorst 1999; Willman and Makisaka 2010). The transferability of interventions from high-income countries that do not share these features, although promising, may be questionable. In low resource setting, there might be a lack of well-functioning institutions within the primary health care and educative systems and thus interventions relying completely on these systems might fail (WHO 2015).

To further advance the prevention of youth violence in Latin America, a region that has been severely affected by this problem during decades, it is important to identify and synthesise evidence from prevention efforts conducted within the region. This will support more informed decision-making by allowing the identification of strategies that have showed the best results under similar contexts. This systematic review, therefore, aims to synthesise evidence on the effectiveness of interventions to prevent violence and crime committed by young people in Latin America. The review focuses on the prevention of interpersonal community violence among youths and does not include other forms of violence such as child maltreatment, intimate partner violence or dating violence.

## Methods

A protocol was prepared in advance by the authors and is available upon request. The review was conducted according to standards from the Preferred Reporting Items for Systematic Reviews and Meta-Analyses statements—PRISMA (Moher et al. 2009) and the Cochrane Collaboration (Higgins and Green 2011). The words programme and intervention are used in an equivalent manner.

### Inclusion and exclusion criteria

We looked for peer-reviewed articles and grey literature in the form of reports, book chapters, conference papers or theses. Studies were included if they: (a) described an intervention to prevent violence among people aged 10–24 years (or the equivalence using school grades); (b) presented quantitative results on the evaluation of an intervention using a variation of study designs such as randomised or non-randomised controlled trials, paired or matched studies, time series, before-after studies with or without comparator arms or any other design based on a quantitative approach. We decided to include any type of design to illustrate the quality of studies conducted in the region; (c) described any type of primary or secondary prevention strategy. Interventions could be implemented at the individual, family, school or community levels and had to be designed to explicitly prevent youth violence or to prevent youth risk behaviours but a reduction in crime, violence, bullying and/or aggression should be stated as a purpose. Participants in the intervention could be any population; (d) outcomes were measures of violence and/or crime such as murders, fighting, aggression, robbery or bullying, both at the individual or community/group level. Outcomes could have been self-reported or reported by others, and needed to have had data on behaviours and not only on related factors such as knowledge or attitudes; e) the intervention was implemented in any country from Central and South America, excluding the Caribbean, Surinam, Guyana and French Guiana.

Studies were excluded if: the manuscript did not provide information on the specific range of age of participants or the educational level targeted, or when the mean age of the youths was below 10 years; the intervention or strategy consisted of a structural intervention that involved the modification to the physical context only; the manuscript did not provide baseline measurement for the main outcomes; or if the intervention consisted exclusively on the incarceration of participants or in sanctions as a consequence of violent behaviour. In other words, we selected studies with a focus on prevention and not on rehabilitation initiatives. We excluded studies presenting outcomes relating to dating, sexual or intimate partner violence.

### Search strategy

A search of the literature was performed by the lead reviewer between February and March 2015 using English as the main language and Spanish for specific databases. Documents in another language were not included considering time and financial constraints for translation into English. An electronic search in academic databases was conducted by title/abstract and descriptors using a comprehensive list of keywords grouped into four concepts: *Population* (adolescents OR young people OR youths OR teenagers, etc.); AND *Intervention* (intervention OR programme OR curriculum OR preventive strategy, etc.); AND *Outcomes* (violence OR antisocial behaviours OR aggression OR crime OR robbery OR fights OR injuries, etc.); AND *Context* (the complete list of countries in Latin America). The list of terms was developed by the lead reviewer and reviewed by other members of the team. The complete list of searched terms is available upon request.

The following databases were explored using English: ASSIA, CINAHL, Child Development and Adolescent Studies, ProQuest Dissertation and Theses A&I, Education Abstracts, Education Journals, ERIC, IBSS, MEDLINE/Pubmed, National Criminal Justice Reference Service Abstracts Database, PsycINFO, SCOPUS, Social Services Abstracts, Sociological Abstracts; and in Spanish: LILACS, Periódica and SCIELO. A sample of the search strategy in ASSIA is presented as online supplementary material. In addition to electronic database searching, a search in the websites of 18 relevant national and international institutions (such as Institute CISALVA; J-PAL; the Center for International Conflict Resolution, Creative Associates International, etc.) was conducted. In this case, we focused only on identifying full-text documents, and we used searches in Google to locate documents when a programme was mentioned in a webpage and no report was provided. Reference list checking and citation searching was also carried out. Year limits were not specified for the search since we aimed to identify all the published papers.

### Study selection and data extraction

Results from the search were downloaded into EndNote X7. Relevant publications were selected based on the titles and abstracts and the full text was retrieved for those papers that met the inclusion criteria or those in which eligibility was not clear. The full text was then used for in-depth screening. We did not make an attempt to retrieve papers when the full text was not available to us online; i.e. books or theses. For each included study, specific information was retrieved using a data extraction sheet piloted a priori to collect data regarding identification and characteristics of the study, intervention description and major findings. The lead reviewer conducted the screening for inclusion of the potential studies and the data extraction, and the final sample of selected manuscripts was confirmed by a second reviewer. Any queries in regards to study inclusion were discussed and decided within the team.

### Quality assessment

The quality of each included study was assessed using an adaptation of the *Quality Assessment Tool for Quantitative Studies* (Effective Public Health Practice Project (EPHPP) 1998), composed of six general components that are assessed by a set of individual items. This tool covers any quantitative study design and it is particularly useful for research related to public health (Thomas et al. 2004). In this review the categories of “High risk of bias”, “Low risk of bias” and “Unclear risk of bias” were used similar to the assessment of risk of bias proposed by the Cochrane Collaboration.

### Data synthesis

Due to the heterogeneity of interventions and settings, a meta-analysis was not feasible and thus the synthesis was conducted in a structured narrative format with the support of tabular supplements (Popay et al. 2006). An assessment of the risk of bias across the cumulative evidence was not performed for the same reasons.

## Results

**Fig. 1:**
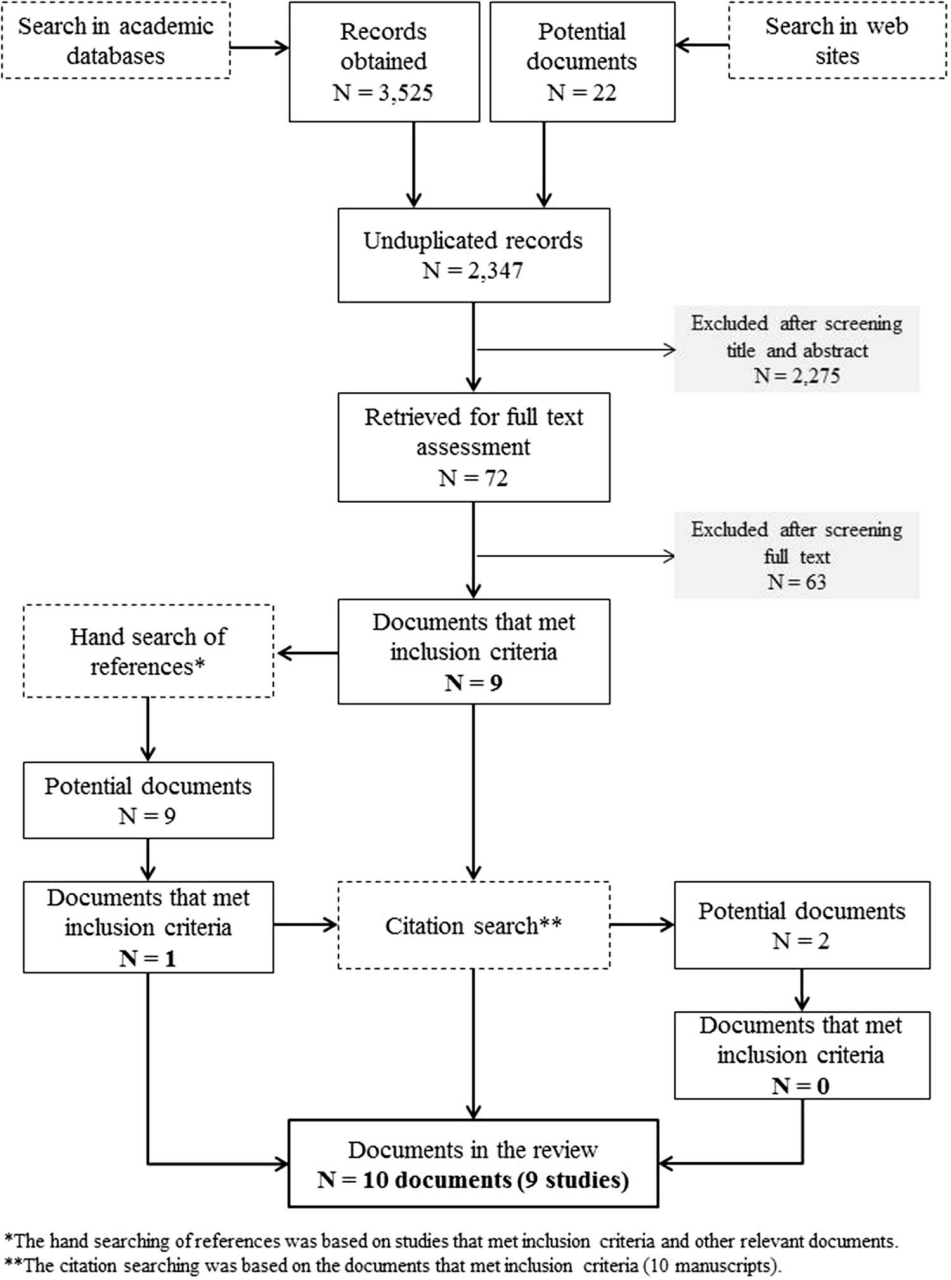
PRISMA flow diagram of the search of literature about youth violence prevention interventions. Latin America, 2015

A total of 3547 records were obtained from the electronic search. The PRISMA diagram in Fig. 1 outlines the study selection process. In total, 10 papers were included that presented the results of nine studies (Berk-Seligson et al. 2014; Berthelon and Kruger 2011; Kenney and Godson 2002; Muñoz-Vallejos and Rosales-Donoso 2008; Pérez et al. 2013; Reyes-Moreno 2011; Silveira et al. 2010; Tijmes and Varela 2008; Varela 2011; Varela et al. 2009). Other documents were consulted to collect details of some interventions (Alves and Arias 2012; Castro and Escribens 2012; Godson and Kenney 2000; Lecannelier et al. 2011; Silveira 2007).

### Description of the studies

A description of the studies and programmes is presented in Table 1. Five studies were conducted in Chile and the others in Brazil, Peru, El Salvador and in the Mexico-US border, mostly in a school setting. The study conducted by Berk-Seligson et al. (2014) was the only one that relied on a clustered randomised controlled design, with data collected from an adult population regarding the presence of youth violence. In addition, two studies used an ecologic design with aggregated measures (official statistics) rather than individual data (Berthelon and Kruger 2011; Silveira et al. 2010). The rest relied on the self-report of youths regarding involvement in violent behaviours, four of which used a before-after design without control group, one a non-randomised controlled trial and another a cross-sectional comparison of two groups. With the exception of the study conducted by Pérez et al. (2013) targeting female students, all focused on both males and females.

**Table 1:** Characteristics of the included studies

	Authors	Country	Study design^a^	Control group	Study setting	Target population	Sample (baseline)	Intervention type	Intervention name	Length	Main contents/activities
1	Berk-Seligson et al. (2014)	El Salvador	Cluster randomised controlled trial	Neighbourhoods without the programme	41 at-risk neighbourhoods from 4 municipalities	Community but focused on youths	2399	Community-based; Multiple Components	Central America Regional Security Initiative (CARSI)	28 months	(a) Social entrepreneurship skills for youths and leaders; (b) Vocational training; (c) Theatre, painting and puppetry; (d) Counseling programs for at youth-at-risk and their families; (e) Grants for school equipment; (f) Youth clubs; (g) Conflict mediation among teachers, students, parents and community leaders; (h) Radio
2	Berthelon and Kruger (2011)	Chile	Ecologic study, time-series analysis	No control	Public schools; municipalities	Students: 9th–12th grade	NA; ecologic design	National school reform	Full-day School Reform	NA	Full-day school reform to increase the amount of time that students spend in school from 32 to 39 h per week (22 % of time increase)
3	Kenney and Godson (2002)	Mexico and US	Non-randomised controlled trial	Schools without the programme	11 schools in urban areas from 2 border cities	Students: 9th grade	814	School-based	School-Based Education to Counter Crime and Corruption	4 months	Class-room based curriculum on: (a) Values, self-Esteem and a culture of lawfulness; (b) Organised crime and corruption; (d) Furthering the rule of law, resistance techniques and what students can do
4	Muñoz-Vallejos and Rosales-Donoso (2008)	Chile	Cross-sectional, two groups	Schools where the programme started recently	6 public schools in 1 urban area	Students: 5th–10th grade	502	School-based; Multiple Components	Programa de Mediación Escolar	12 months^b^	(a) Modifications to school rules; (b) Implementation of 14 weeks “for a good coexistance in school”; (c) Training to teachers, Principals and students/peers in mediation (200 h of training)
5	Pérez et al. (2013)	Chile	Before-after study (panel of schools/grades)	No control	1 school in urban area	Female Students: 4th–12th grade	320	School-based; Multiple Components	Vínculos	20 months	(a) Promotion of the programme; (b) Meetings with teachers, students and parents; (c) Students design of programme name and logo; (e) Newsletter to parents, teachers and students; (f) Skills development in the classroom; (g) Mailbox for complaints of bullying; (h) Detection and monitoring of cases of bullying
6	Reyes-Moreno (2011)	Peru	Before-after study (panel of schools/grades)	No control	Public schools in 4 regions	Students: 5th–10th grade	537	Family based	Familias Fuertes Amor y Límites	1 month, 3 weeks	(a) Groups for parents; (b) Groups for adolescents; (c) Activities for parents-adolescents; (d) Skills development for parents monitoring, listening and empaty; (d) Skills development for avoidance of peer pressure and risks; (e) Promotion of parents-adolescents communication
7	Silveira et al. (2010)	Brazil	Ecologic study, time-series analysis	Violent and non-violent favelas without the programme	1 urban at-risk area (violent and non-violent favelas)	Community but focused on youths	NA; ecologic design	Community-based; Multiple components	Staying Alive [Fica Vivo]	52 months^b,c^	(a) Promotion of the programme; (b) Mobilisation of police (search and seisure of arms, search and arrest warrants, police occupation of trafficking places); (c) Policing of special risk areas; (d) Workshops and events (sporting, cultural, citizenship, health and professional) to youths 20 h per week; (e) Working groups to solve local problems (health, education and productive involvement)
8	Varela et al. (2009), Tijmes and Varela (2008)	Chile	Before-after study (panel of schools/grades)	No control	3 schools in 1 urban area	Students: 5th–12th grade	2007	School-based; multiple components	Programa Paz Educa	24 months^b^	(a) Improvements to physical environment in school; (b) Training to teachers; (c) Key messages and skills development in classroom; (d) Individual counselling to students with problematic behaviours; (e) Meetings with parents
9	Varela (2011)	Chile	Before-after study (panel of schools/grades)	No control	4 schools in 1 urban area	Students: 5th–12th grade	677	School-based; Multiple components	Recoleta en Buena	24 months^a^	(a) Improvements to physical environment in school; (b) Training to teachers; (c) Skills development in classroom; (d) Individual counselling to students with problematic behaviours; (e) Meetings with parents and community key actors to strengthen the link between the school and health services/other social programmes

A systematic review of interventions to prevent youth violence. Latin America, 2015 (Countries from Latin America, excluding the Caribbean, Surinam, Guyana and French Guiana; primary or secondary preventive interventions)

*NA* Not applicable

^a^According to the authors of this review

^b^No information is provided on the exact number of months or weeks of implementation

^c^The programme decreased the activities after 7 months and started after 12 months. The activities focused on youths ran from month 22–53

Two important considerations should be mentioned. First, the studies conducted by Berk-Seligson et al. (2014) and Silveira et al. (2010) described programmes to prevent violence in the general population. Both were included since the interventions had a strong focus on youths. In the case of Berk-Seligson et al. (2014), outcomes related to youths were prioritised as well as homicides and other outcomes such as robberies are not reported. Second, the study conducted by Berthelon and Kruger (2011) assessed the effects of a reform to extend the hours at school. While this would be a structural intervention, it is one of the few analysing the effects on juvenile crime of one such programme; more importantly, more time in the school means more opportunities to increase academic achievements and human capital (Berthelon and Kruger 2011; Patall et al. 2010), and thus its inclusion was warranted.

### Description of the programmes

Two studies described wide community-based programmes ranging from 28 to 52 months in length (Berk-Seligson et al. 2014; Silveira et al. 2010) and one was a family based intervention with a length of one month three weeks (Reyes-Moreno 2011). The rest were school-based implemented with a range of four to 24 months. The majority of the programmes comprised multiple components or strategies such as training to teachers, classroom-centred activities or activities within the school. Six of the programmes involved family members and three involved community key actors.

The school-based programme “Paz Educa” stands out because it was evaluated by Varela et al. (2009) and by Tijmes and Varela (2008), but was adapted, implemented and evaluated again by Varela (2011) and later by Pérez et al. (2013). It is based on principles of positive behaviour support and prevention though environmental design. *“Familias Unidas”* presented by Reyes-Moreno (2011) is a family based strategy promoting quality relationships and has been widely used in South America. Muñoz-Vallejos and Rosales-Donoso (2008) evaluated “Programa de Mediación Escolar”, an intervention promoting mediational skills and conflict resolution; while Kenney and Godson (2002) evaluated “Education to Counter Crime and Corruption”, a classroom structured curriculum focused on the prevention of corruption. The *CARSI* programme evaluated by Berk-Seligson et al. (2014) comprised several activities in the community including participation from the police, school officers and religious leaders. It has been implemented in different countries of Central America. The community-based strategy “Staying Alive” was designed to reduce homicides on high risk favelas in Brazil and included participation of police and workshops for young people (Silveira et al. 2010). Lastly, Berthelon and Kruger (2011) evaluated a structural intervention to extend the time that students stay at school from 32 to 39 h per week.

### Methodological quality of studies

The risk assessment for each study is presented in Table 2. In six studies, the selection of sampling units was not conducted in a systematic manner and detailed information on the selection process was missing (Kenney and Godson 2002; Muñoz-Vallejos and Rosales-Donoso 2008; Pérez et al. 2013; Reyes-Moreno 2011; Tijmes and Varela 2008; Varela 2011; Varela et al. 2009). In general, the reporting of confounders was poor, with only two studies acknowledging the use of controlled analysis to account for potential confounders (Berk-Seligson et al. 2014; Berthelon and Kruger 2011). Considering the ranking of study designs proposed by the quality assessment tool, the majority of the studies were rated as “high risk” with only two studies using low risk designs (Berk-Seligson et al. 2014; Kenney and Godson 2002). The two ecologic studies (Berthelon and Kruger 2011; Silveira et al. 2010) were considered to be of low risk regarding blinding of participants, since measurements on the outcomes were not based on self-reporting but on official data available. In the rest, the outcomes assessors were aware of the intervention status and thus a potential risk is present. There was a lesser risk of bias associated with data collection since most of the studies were based on previously validated scales or surveys. In general, information on drop-outs was not reported.

**Table 2:** Risk of bias of the included studies

	Authors	Selection bias	Study design	Confounders	Blinding	Data collection	Withdrawals/dropouts
1	Berk-Seligson et al. (2014)	Low risk	Low risk	Low risk	High risk	Low risk	Not applicable
2	Berthelon and Kruger (2011)	Not applicable	High risk	Low risk	Low risk	Low risk	Not applicable
3	Kenney and Godson (2002)	Unclear risk	Low risk	Unclear risk	High risk	Unclear risk	Unclear risk
4	Muñoz-Vallejos and Rosales-Donoso (2008)	Unclear risk	High risk	Unclear risk	High risk	Unclear risk	Not applicable
5	Pérez et al. (2013)	Unclear risk	High risk	Unclear risk	High risk	Low risk	Unclear risk
6	Reyes-Moreno 2011	Unclear risk	High risk	Unclear risk	High risk	Low risk	Unclear risk
7	Silveira et al. (2010)	Not applicable	High risk	Unclear risk	Low risk	Low risk	Not applicable
8	Varela et al. (2009), Tijmes and Varela (2008)	Unclear risk	High risk	Unclear risk	High risk	Unclear risk	Low risk
9	Varela (2011)	Unclear risk	High risk	Unclear risk	High risk	Low risk	Low risk

A systematic review of interventions to prevent youth violence. Latin America, 2015 (using an adaptation of the Quality Assessment Tool for Quantitative Studies)

### Effects of the programmes

Table 3 presents detailed results by study while Table 4 presents a summary of the findings. Most of the studies presented evidence of a positive and significant effect on the prevention of youth violence while three document some form of a negative effect (Kenney and Godson 2002; Reyes-Moreno 2011; Tijmes and Varela 2008; Varela et al. 2009). In El Salvador, a reduction in the perception of the presence of murders by 40 % was documented after 29 months of interventions (*p* ≤ 0.05) (Berk-Seligson et al. 2014). Similarly in Brazil a reduction of more than 60 % in the average number of monthly homicides was observed after implementation of the programme; however, the reductions observed during the period in which the prevention activities focused on youth were not different to the ones obtained after the first months when the programme was not exclusively focused on youths (Silveira et al. 2010). After implementation of the school reform, the number of violent crimes committed by young people (homicides, assaults, rape and offenses) decreased by 11 % in Chile (*p* ≤ 0.05) (Berthelon and Kruger 2011).

**Table 3:** Results of the studies

ID	Authors	Follow-up	Outcome	Effect^a^		*p* value	Test or analysis	Unit of observation	Comments
1	Berk-Seligson et al. (2014)	29 months	Perception of homicides in the neighbourhood	−	40 %	*p* ≤ 0.05	Multilevel model	Adult population	Adjusting for co-variates and also by sampling design
			Perception of youth in gangs within the neighbourhood	−	14 %	*p* ≤ 0.05			
			Perception of gang fights in the neighbourhood	−	12 %	*p* ≤ 0.05			
2	Berthelon and Kruger (2011)	48 months	Violent crimes (assaults, homicides, rape and offenses) committed by juveniles (among 14-17 years old)	−	11 %	*p* ≤ 0.05	Fixed-effects regression	Municipality	Adjusting for co-variates
			Property juvenile crimes (among 14-17 years old)	−	24 %	*p* ≤ 0.01			
			Total juvenile crimes (among 14-17 years old)	−	19 %	*p* ≤ 0.01			
3	Kenney and Godson (2002)	4 months	Involvement in deviant activities (theft, vandalism and disorderly conduct), San Diego	+	NS	NS	NS	Students	Outcome reported narratively; no *p* values mentioned
			Involvement in deviant activities (theft, vandalism and disorderly conduct), Tijuana	No differences		NS			
4	Muñoz-Vallejos and Rosales-Donoso (2008)	12 months	Perception of fights as an event occurring at school	−	17 %	*p* ≤ 0.01	*t* test	Students	Not all the outcomes are presented as % or with a level of significance
			Perception of threats as an event occurring at school	−	9 %	*p* ≤ 0.05			
			Perception of fights or threats in school per week	−	0.86 (µ)	*p* ≤ 0.05			
5	Pérez et al. (2013)	20 months	Witnessed bullying in the school	−	0.99 (µ)	*p* ≤ 0.01	*t* test	Students	Measured using scales. When using items from the witness scale there were 2 significant reductions
			Committed/experienced serious bullying	−	0.02 (µ)	*p* > 0.1			
			Committed bullying	−	0.09 (µ)	*p* > 0.1			
6	Reyes-Moreno (2011)	2 months	Involvement in antisocial behaviours	−	0.40 (µ)	*p* ≤ 0.01	Signed-rank test	Students	Measured using scales
			Involvement in intentional aggression	+	0.30 (µ)	*p* ≤ 0.01			
			Involvement in delinquency	+	0.10 (µ)	*p* ≤ 0.01			
7	Silveira et al. (2010)	32–36 months	Average of monthly homicides (*vs* violent favelas)	−	69 %	NS^b^	Generalised linear model	Areas/favelas	All comparsions were significant when comparing with baseline values but were not different when comparing within the follow-ups
			Average of monthly homicides (*vs* non-violent favelas)	−	64 %	NS^b^			
			Average of monthly homicides (*vs* neighborhoods)	−	60 %	NS^b^			
		53–84 months	Average of monthly homicides (*vs* violent favelas)	−	61 %	NS^b^			
			Average of monthly homicides (*vs* non-violent favelas)	−	52 %	NS^b^			
			Average of monthly homicides (*vs* other neighborhoods)	−	69 %	NS^b^			
8	Varela et al. (2009); Tijmes and Varela (2008)	24 months	School 1 Perception of fights in the school	−	10 %	*p* ≤ 0.05	*t* test	Students	Results for school 2 not clear; not statistical significance provided for all the mentioned outcomes
			School 1 Perception of robbery in the school	−	8 %	*p* ≤ 0.05			
			School 1 Perception of intentional damages to school	−	12 %	*p* ≤ 0.05			
			School 1 Perception of threats, students to teachers	−	8 %	NS			
			School 1 Perception of aggression, students to teachers	−	7 %	NS			
			School 1 Perception of insults, students to teachers	−	11 %	NS			
			School 2 Perception of robbery in the school	+	19 %	*p* ≤ 0.05			
			School 3 Perception of threats among students	+	14 %	*p* ≤ 0.05			
			School 3 Perception of insults among students	+	9 %	NS			
			School 3 Perception of robbery in the school	+	17 %	NS			
9	Varela (2011)	29 months	Witnessed antisocial behaviours	−	0.16 (µ)	*p* ≤ 0.01	*t* test	Students	Measured using scales; not statistical significance provided for all the mentioned outcomes
			Witnessed violence between peers	−	0.17 (µ)	*p* ≤ 0.01			
			Witnessed violence, students to adults	−	0.34 (µ)	*p* ≤ 0.01			
			Committed violence to peers	−	0.14 (µ)	*p* ≤ 0.01			
			Committed serious violent acts to peers or to teachers	−	0.03 (µ)	*p* > 0.1			

A systematic review of interventions to prevent youth violence. Latin America, 2015

*NS* not specified because results were described narratively but not numerical

^+^Increase over time or higher proportion/values among the intervention group. Effects presented as percentage or difference in means when stated

^a^Reduction over time or lower proportion/values among the intervention group

^b^Significant results using confidence intervals but *p* values not reported

**Table 4:** Synthesis of the results

Outcome	Number of studies that:		
	Measured it	Documented a reduction^a^	Documented an increase^a^
Homicides (official records or perception of occurrence)^b^	2	2	0
Youth engament in violent behaviour (fights, bullying, antisocial behaviours), self-reported	3	2	1
Youth engagement in crime, deviant behaviours, vandalism, etc., self-reported	2	0	1^c^
Presence of youth violence within the school/community, as reported by others	6	6	1

Interventions to prevent youth violence. Latin America, 2015

^a^Studies that documented a significant result with *p* ≤ 0.05 in at least one measurement

^b^In addition, one study measured juvenile violent crimes including but not limited to homicides and documented a statistical significant reduction post-intervention

^c^The second study also documented an increase but not information on *p* values were provided

Considering the self-report on the involvement in violence, crime or bullying, mixed results were found. In Chile, involvement in violence decreased after 29 months following a school-based intervention (*p* ≤ 0.01), although no statistical significance was found for serious violent acts (Varela 2011). Similarly, in another study also from Chile no statistical differences were found after 20 months (*p* > 0.1) (Pérez et al. 2013). In the Mexico-U.S. border it was reported an increase in deviant behaviours in one school but no differences in another after four months of a classroom-based curriculum (Kenney and Godson 2002). In Peru, a reduction in involvement in antisocial behaviour was found after two months, but an increase in intentional aggression following a family based intervention (*p* ≤ 0.01) (Reyes-Moreno 2011). Regarding the perception of violence or crime committed by other youths in the school or community, the studies with Chilean students documented a reduction after 12 months in both fights and threats (*p* ≤ 0.05) (Muñoz-Vallejos and Rosales-Donoso 2008), in bully after 20 months (*p* ≤ 0.01) (Pérez et al. 2013) and antisocial behaviours and violence after 29 months (*p* ≤ 0.01) (Varela 2011). One study found after 24 months a reduction in the perception of fights and damages in one school but an increase in threats and robberies in two other schools (*p* ≤ 0.05) (Tijmes and Varela 2008; Varela et al. 2009).

## Discussion

This review was conducted to assess the evidence of the effectiveness of interventions to prevent violence in young people from Latin America. As in many other developing regions, in Latin America the question of *what programmes work?* (Nation et al. 2003), is still an unanswered one for the case of youth violence. In this sense, the systematic review presented here is one of the first focused within the region.

In relation to the effectiveness of the programmes, it can be stated that most of them documented positive effects; however, the evidence is still insufficient. The most stimulating findings were in relation to reductions in homicides, since two studies assessed this outcome and both documented a reduction; one of them using a clustered randomised controlled design (Berk-Seligson et al. 2014) and the other a time-series data analysis using registries from the police (Silveira et al. 2010). These studies were the ones showing more methodological rigour and also they were the only ones assessing the effects of wide community-based initiatives. In addition, a study assessing the effects on a school reform to extend the hours at school also documented a reduction in juvenile violent crimes including homicides according to official registries within the municipality (Berthelon and Kruger 2011).

There seems to be also promising evidence of the effectiveness of the programmes when measuring the perception of other peers or adults about the presence of youth violence within the community. More than half of the studies assessed this type of outcome and all documented at least one positive change after the intervention. On the other hand, contradictory evidence was found considering the self-report of youths regarding participation in violent acts or crime. This may be related to differences in the way of measuring the outcomes. In the case of homicides, more consistency may exist because homicide is a more objective indicator; however, there is not a unique definition for violence. Some studies measured violence committed against other peers, while others measured involvement in antisocial behaviours, participation in bullying or serious bullying. This inconsistency can also be related to the fact that most of these outcomes come from self-report. Considering violence as an undesirable behaviour, the self-report of participants involves the risk of response bias. In this sense, it is motivating that many studies documented a reduction on participants’ perception of violence committed by peers, another indicator of the presence of youth violence. Overall, it can be said that these results are optimistic.

As mentioned before, heterogeneity among the studies and programmes makes impractical to compare the results across studies. Because of this, it is not possible to provide an accurate answer to the question of what are the programmes that work best in the prevention of youth violence. However, some lessons can be mentioned. Most of the programmes included different activities and multiple components and those assessing a single intervention documented inconsistent results. Previous international evidence shows that no clear consensus exists regarding the benefits of multicomponent versus single programmes for the prevention of youth violence (Matjasko et al. 2012). Judging by the evidence described here, there is more evidence in favour of multicomponent strategies and it could even be stated that multicomponent community-based interventions that involve different levels of key actors (i.e. the police, community leaders, families) provided the most promising findings with a reduction in homicides. However, we only included two community-based programmes. More importantly, it is not possible to draw conclusions about successful elements since evidence of impact was not presented for each component of the interventions.

Recently, one scooping review focused on Latin America and the Caribbean was published describing evaluations of youth violence preventive interventions (Moestue et al. 2013). The previous review is different to the one presented here in that it considers all types of interpersonal violence, including sexual and domestic; it includes studies from the Caribbean; it focuses on randomised controlled trials exclusively; includes ongoing studies; and was based on a comprehensive search of grey literature but not peer-reviewed literature (Moestue et al. 2013). Similarly to Moestue review, we found that most of the programmes evaluated a school-based intervention, meaning that the evidence is strongest for this type of programme. While it is important to recognise the role of school for the implementation of these programmes, it cannot go unnoticed that the most vulnerable group, i.e. young people that are not in school, are not being targeted by these interventions. Also important is to acknowledge that the most severe form of violence rarely occurs within schools (Basch 2011; Hahn et al. 2007).

In 2015, the World Health Organization published a synthesis regarding global evidence from interventions to prevent youth violence. According to such report, the most promising interventions to prevent perpetration of youth violence are strategies implemented at the community level, including community-police partnerships, reducing access to firearms or promoting drug control programmes (WHO 2015). Thus, it seems that in line with international efforts, community-focused strategies have proven to be effective also within the Latin American region, although the evidence is still limited.

Regarding school-based initiatives, the WHO global report suggests that life and social skills development and bullying prevention programmes are promising strategies. However, the evidence coming from other forms of school-based programmes is less clear regarding their effectiveness (WHO 2015). In our review, the results from school-based interventions are mixed since most of the interventions documented positive reductions on violence; however, some negative trends were also observed. On one hand, our results might reflect the impact of less rigorously designed studies since most of the evaluations relied on before-after designs; on the other hand, we need to consider the existence of a context in which education quality is poor and might affect the results of prevention programmes. According to a recent meta-review of international studies assessing the effectiveness of interventions to prevent youth violence, the most common form of interventions in the world are school-based and family based, but the latter are the ones with stronger evidence of effectiveness (Matjasko et al. 2012). The WHO report highlights that parenting strategies seems to be another form of promising interventions (WHO 2015); however, in this review we did not find strong evidence coming from family based studies as only one study assessing this type of intervention was included. The evaluation of parent or family based strategies is needed.

Some other important lessons can be mentioned. The programme *Staying Alive* in Brazil and the *CARSI* initiative in El Salvador are proof that large and complex interventions involving community members can successfully operate in parallel with efforts that involve the police, community key actors and even religious leaders. Also, the large evidence coming from school-based programmes shows how such programmes can be easier to implement and adapt to different settings, while the national school reform analysed provides an example of how changes in the school system positively affects the communities outside the school setting. Countries in Latin America could take advantage of these studies; for example from the wide experience of Chile where school-based interventions to reduce school-violence have been largely implemented. This review also shows that most of the programmes omitted gender issues although youth violence has consistently been known to be highly elevated among males, with lesser rates for females. Prevention efforts need to recognise this when aiming to prevent violent behaviours in men and women.

Some limitations are discussed next. Only nine studies were found. A limited number of studies were expected, though the lack of high-quality research is surprising. While it was anticipated that the inclusion of non-experimental designs would raise concerns, almost all of the studies can be judged as presenting bias and inadequate reporting. Considering the quality of the individual studies, the strength of the evidence summarised in this review can be considered weak. Admittedly, more rigorous criteria could have been applied for the inclusion of studies, but such rigour would have meant the location of fewer studies. For the purposes of this review, it was important to assess the quality of the studies that are being conducted. It is interesting to note that most of the studies were published within the last five years. This may represent a trend about evaluation studies in Latin America (Moestue et al. 2013). Also, it is important to recognise the efforts of researchers in assessing these interventions since evaluation studies in resource-limited-settings are costly. The risk associated with the use of methodological diversity and low-quality research is acknowledged and thus findings should be interpreted with caution.

Another limitation is that manuscripts in Portuguese were not included. This is important because many research has been conducted in Brazil given the high levels of youth violence; however, not all research might be published in English. In addition, we did not try to retrieve papers that were not published online and we did not make an attempt to contact relevant authors. Thus, the possibility exists that some other studies that have been conducted are not included in this review.

Some implications for future research are derived. This synthesis exhibits the need for rigorously designed evaluation studies in the region. Studies assessing the effects of interventions should take into account socio-demographic aspects and other potential confounders during data analysis and could use sophisticated statistical techniques that could aid in the management of methodological concerns such as multilevel models, interactions, matching procedures, analyses for complex sampling or procedures for missing data. There is a clear need for reliable data as well as standardised instruments and indicators to measure youth violence (Moestue et al. 2013). Authors should make an effort to report detailed and complete procedures and results. The fact that we found a larger number of school-based interventions might reflect that studies within schools are easier to implement and control. Studies in which parents or other key community players are involved face the challenge of having access to the target population, maintaining their permanence in the study and being able to identify and control factors that might affect how a programme is implemented and evaluated. Researchers need to carefully consider these aspects to choose the appropriated design when evaluating interventions different to school-based initiatives, as evidence from other types of strategies is needed. The more complex the programme, the more complex the evaluation design.

To conclude, this review identified, appraised and synthesised the evidence regarding the evaluation of programmes to prevent youth violence, crime and bullying in Latin America. The findings show that most of the interventions had promising results on the prevention of youth violence, particularly regarding reductions in homicides and on the perception of the presence of violent acts committed by others. While community-based programmes showed more consistency regarding the effectiveness to prevent violence, the evidence comes only from two studies. Overall, the evidence is still limited in terms of quantity and relies mostly on non-experimental designs. However, this synthesis is a good starting point and could contribute to the process of decision-making regarding investments in interventions; a critical matter in resource-limited-settings.

## Electronic supplementary material

Below is the link to the electronic supplementary material.

Supplementary material 1 (DOC 39 kb)
