# Supplementary material for: Interventions to prevent youth violence in Latin America: a systematic review
Source: Int J Public Health. 2016 Oct 20;62(1):15–29. doi: 10.1007/s00038-016-0909-6 (PMC5288433; doi:10.1007/s00038-016-0909-6)
Supplement: Supplementary file 1 — Supplementary material 1 (DOC 39 kb) [file 38_2016_909_MOESM1_ESM.doc]

**International Journal of Public Health**

**Interventions to prevent youth violence in Latin America: A systematic review**

**Atienzo, Erika E.^1^*, Baxter, Susan K.^1^, Kaltenthaler, Eva.^1^**

^1^ School of Health and Related Research, University of Sheffield, Sheffield, UK.

Correspondence: e.atienzo@sheffield.ac.uk

**Electronic supplementary material. Example of the search strategy in Applied Social Sciences Indexes and Abstracts (ASSIA) database.**

# 1. Concept: **Population**

AB,TI("Adolescents" OR "Boys" OR "Children" OR "Girls" OR "Minors" OR "Pupils" OR "Teens" OR "Teenagers" OR "School Children" OR "Students" OR "Youngsters" OR "Young Adults" OR "Young People" OR "Youths") OR SU.EXACT("Adolescents" OR "Boys" OR "Adolescent Boys" OR "Young Boys" OR "Children" OR "Girls" OR "Adolescent Girls" OR "Young Girls" OR "Minor" OR "Pupils" OR "Teenagers" OR "Students" OR "Young Adults" OR "Young People" OR "Young Men" OR "Young Women" OR "Youth")

# 2. Concept: **Intervention**

AB,TI("Intervention[*1]" OR "Curriculum[*1]" OR "Program[*1]" OR "Programme[*1]" OR "Preventive Intervention[*1]" OR "Preventive Curriculum[*1]" OR "Preventive Program[*1]" OR "Preventive Programme[*1]" OR "Preventive Strategy" OR "Preventive Strategies" OR "Preventive Project[*1]" OR "Curriculum Evaluation" OR "Program Evaluation" OR "Programme Evaluation" OR "Program Effectiveness" OR "Programme Effectiveness" OR "Intervention Evaluation") OR SU.EXACT("Intervention" OR "Curriculum" OR "Programmes" OR "Preventive Programmes" OR "Preventive Strategies")

# 3. Concept: **Outcomes**

AB,TI("Aggression" OR "Aggressiveness" OR "Attack[*1]" OR "Assault[*1]" OR "Bullying" OR "Crime[*1]" OR "Fight[*1]" OR "Homicide[*1]" OR "Killing[*1]" OR "Murder[*1]" OR "Injuries" OR "Lesions" OR "Offense[*1]" OR "Violence" OR "Interpersonal Violence" OR "Aggressive Behavio*r[*1]" OR "Criminal Behavio*r[*1]" OR "Violent Behavio*r[*1]") OR SU.EXACT("Aggression" OR "Assault" OR "Bullying" OR "Crime" OR "Violent Crime" OR "Fighting" OR "Street Fighting" OR "Homicide" OR "Killing" OR "Murder" OR "Injuries" OR "Lesions" OR "Criminal Injuries" OR "Offending" OR "Criminal Offences" OR "Violence" OR "Street Violence" OR "Community Violence" OR "Alcohol Related Violence" OR "Criminal Behaviour")

# 4. Concept: **Context**

AB,TI("Argentina" OR "Belize" OR "Bolivia" OR "Brazil" OR "Chile" OR "Colombia" OR "Costa Rica" OR "Ecuador" OR "El Salvador" OR "Guatemala" OR "Honduras" OR "Mexico" OR "Nicaragua" OR "Panama" OR "Paraguay" OR "Peru" OR "Uruguay" OR "Venezuela" OR "South America" OR "Central America" OR "Latin America" OR Argentine[*1] OR "Belizean[*1]" OR "Bolivian[*1]" OR "Brazilian[*1]" OR "Chilean[*1]" OR "Colombian[*1]" OR "Costa Rican[*1]" OR "Ecuador?an[*1]" OR "Salvador*an[*1]" OR "Guatemalan[*1]" OR "Honduran[*1]" OR "Mexican[*1]" OR "Nicaraguan[*1]" OR "Panamanian[*1]" OR "Paraguayan[*1]" OR "Peruvian[*1]" OR "Uruguayan[*1]" OR "Venezuelan[*1]" OR "South American[*1]" OR "Central American[*1]" OR "Latin American[*1]") OR SU.EXACT("Argentina" OR "Belize" OR "Bolivia" OR "Brazil" OR "Chile" OR "Colombia" OR "Costa Rica" OR "Ecuador" OR "El Salvador" OR "Guatemala" OR "Honduras" OR "Mexico" OR "Nicaragua" OR "Panama" OR "Paraguay" OR "Peru" OR "Uruguay" OR "Venezuela" OR "South America" OR "Central America" OR "Latin America" OR "Bolivian People" OR "Brazilian People" OR "Chilean People" OR "Colombian People" OR "Ecuadorean People" OR "Salvadorean People" OR "Guatemalan People" OR "Mexican People" OR "Nicaraguan People" OR "Peruvian People" OR "South American People" OR "Central American People" OR "Latin American People")

**Number of hits: 72**
